# Different Subsets of T Cells, Memory, Effector Functions, and CAR-T Immunotherapy

**DOI:** 10.3390/cancers8030036

**Published:** 2016-03-15

**Authors:** Vita Golubovskaya, Lijun Wu

**Affiliations:** Promab Biotechnologies, 2600 Hilltop Drive, Suite 320, Richmond, CA 94803, USA

**Keywords:** chimeric antigen receptor (CAR), immunotherapy, cancer, CD4 T cells, CD8 T cells

## Abstract

This review is focused on different subsets of T cells: CD4 and CD8, memory and effector functions, and their role in CAR-T therapy––a cellular adoptive immunotherapy with T cells expressing chimeric antigen receptor. The CAR-T cells recognize tumor antigens and induce cytotoxic activities against tumor cells. Recently, differences in T cell functions and the role of memory and effector T cells were shown to be important in CAR-T cell immunotherapy. The CD4^+^ subsets (Th1, Th2, Th9, Th17, Th22, Treg, and Tfh) and CD8^+^ memory and effector subsets differ in extra-cellular (CD25, CD45RO, CD45RA, CCR-7, L-Selectin [CD62L], *etc.*); intracellular markers (FOXP3); epigenetic and genetic programs; and metabolic pathways (catabolic or anabolic); and these differences can be modulated to improve CAR-T therapy. In addition, CD4^+^ Treg cells suppress the efficacy of CAR-T cell therapy, and different approaches to overcome this suppression are discussed in this review. Thus, next-generation CAR-T immunotherapy can be improved, based on our knowledge of T cell subsets functions, differentiation, proliferation, and signaling pathways to generate more active CAR-T cells against tumors.

## 1. Introduction

Cellular immunotherapy, such as CAR-T, a therapy with T cells expressing antibody-based chimeric antigen receptor targeting tumor antigen, is an effective therapy against different types of hematological malignancies and also against solid cancers [1,2]. CAR-T (initially called a T body), meaning a T cell expressing an antigen-specific or antibody-based chimeric receptor with antibody specificity and T-cell effector or regulatory function, was first described in the 1980s by Eshhar and his colleagues at the Weizmann Institute of Science in Israel [3].

**Figure 1:**
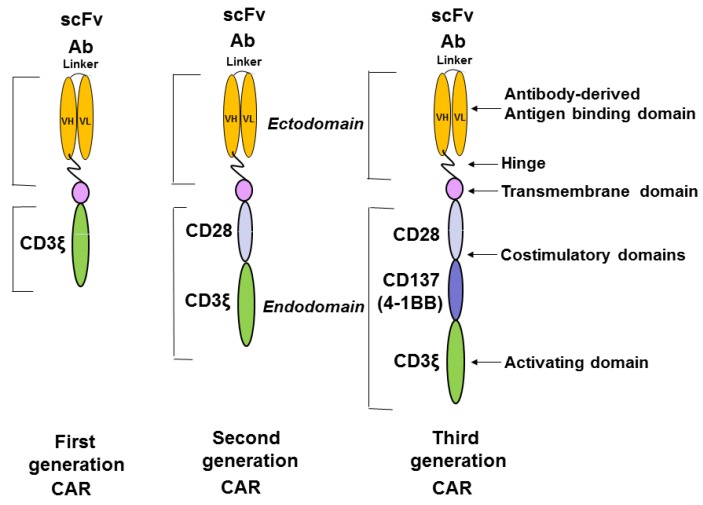
The structure of chimeric antigen receptor construct. The first, second, and third generation of CAR constructs are shown. The first generation of CAR has only an activation domain; the second generation of CAR has one activation domain and one co-stimulatory domain; and the third generation of CAR has one activation domain and two co-stimulatory domains. The ectodomain consists of antibody-derived antigen binding scFv (single chain variable fragment) with the variable fragment of heavy chain, VH, and the variable fragment of light chain VL, which are connected with a linker. The hinge region connects ScFv with the transmembrane domain. The endodomain consists of the co-stimulatory domains (CD28; CD137 or 4-1BB) and the activating domain: CD3 zeta.

CAR combines a single chain variable fragment (scFv) of antibody that drives specificity against tumor antigens [4]. The scFv consists of variable light (VL) and heavy (VH) chains of antibody fused in frame with the linker. The CAR has a hinge, and transmembrane domains, co-stimulatory domains (CD28, CD137 (4-1BB), or other), and activation domain CD-3 zeta (Figure 1). The first generation of CAR had one CD3 domain; the second generation of CAR had an activation domain and one co-stimulatory domain; and the third generation of CAR had one activation and two co-stimulatory domains, as shown in Figure 1. Once CAR-T binds tumor antigen, the T cell proliferation and expansion are activated, with T cell cytotoxic functions causing tumor cell death [1].

Recent reports highlighted the importance of an analysis of the variations between a T cell subset’s functions (memory and effector) and the individual patient’s T cell profile in the efficacy of CAR-T cell immunotherapy [5,6]. For example, recently T memory stem cells (T _SCM_) from a CD45RA^+^ T population with a high expression of CD62L, CD95, and CCR-7 were shown to be more persistent and more effective against tumors than T central memory cells [7]. The authors suggest that the stemness of anti-tumor T cells can increase the high potential of immunotherapy [7]. The detailed mechanisms of T cell subset differentiation, T cell stem-like, memory, and effector functions is important for increasing the efficacy of CAR-T anti-cancer therapy. The comparison of CD4^+^CAR-T and CD8^+^CAR-T cells and their anti-tumor activities, as shown by [8] may improve the design and manufacture of a next-generation CAR-T cell with higher anti-cancer efficacy. Another report demonstrated that combining the most effective subsets of CD8^+^ and CD4^+^ CD19-expressing CAR-T cells resulted in a synergistic anti-tumor effect *in vivo* [9].

Thus, the present review highlights data on the role of different subsets of T cells: CD4^+^ and CD8^+^ cell subsets and differentiation; memory and effector T cell functions; extracellular T cell markers; genetic, epigenetic, and metabolic signaling pathways of T cells and focuses on their role in CAR-T cellular immunotherapy and provides perspectives on improving CAR-T immunotherapy.

## 2. CD4 Cell Subsets

**Figure 2:**
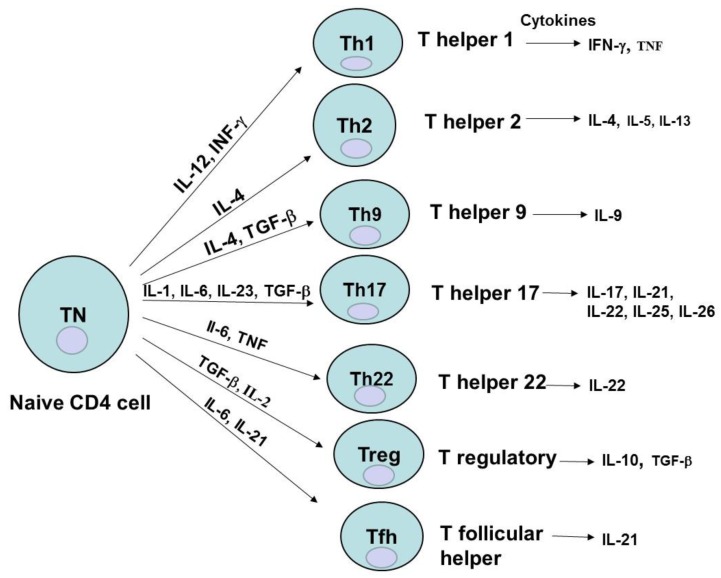
Different CD4^+^ T cell subsets. The different CD4^+^ subsets are generated from the naive T cells by the different cytokines. Each CD4^+^ subset produces a different type of interleukins.

T cells mature in the thymus, express TCR (T cell receptor), and can express either CD8 glycoprotein on their surface and are called CD8^+^ T cells (cytotoxic) or CD4 glycoprotein and are then called CD4 cells (helper T cells). CD4^+^ cells differentiate into different subsets: Th (T helper)1, Th2, Th9, Th17, Th22, Treg (regulatory T cells), and Tfh (follicular helper T cells), which are characterized by different cytokine profiles (Figure 2) [10]. These different CD4^+^ subsets play a critical role in the immune and effector response functions of T cells [10]. All CD4^+^ Th subsets are differentiated from naive CD4^+^ T cells by specific cytokines: Th1 by IL-12 and IFN-γ (pro-inflammatory cytokine, with multiple roles such as increase of TLR (Toll-like receptor), induction of cytokine secretion or macrophage activation); Th-2 by IL-4; Treg by IL-2 and TGF-beta (Figure 2). And each Th subset releases specific cytokines that can have either pro- or anti-inflammatory functions, survival or protective functions. For example, Th1 releases IFN-γ and TNF; Th2 releases IL-4 (an important survival factor for B-type lymphocytes), IL-5 and IL-13; Th9 produces IL-9; Treg secretes IL-10 (a cytokine with an immunosuppressive function, maintaining expression of FOXP3 transcription factor needed for suppressive function of Treg on other cells [11]) and TGF-β; Th17 produces IL-17 (a cytokine playing an important role in host defense against bacteria, and fungi) [10] (Figure 2).

Several reports demonstrated differential roles of different types of cytokines released by CD4^+^ subsets. Th1 and Th2 CD4^+^ T cell subset cytokines were shown to drive different types of cytotoxicity generated by the second generation of CD28-containing CAR-T [12]. Short-term toxicity was observed with high levels of Th1 cytokines, while high doses of Th2 type cytokines generated chronic autocytotoxicity in animals that received second generation CD19-specific CAR-T that should be considered during developing CAR-T therapy [12]. CAR-T cells engineered to deliver inducible IL-12 modulated tumor stroma to destroy cancer [13]. IL-12 release by engineered CAR-T cells increased anti-cancer activity by recruiting macrophages [14]. IL-12 released by CAR-T also induced reprogramming of suppressive cells, reversing their inhibitory functions [13] suggesting its evaluation in clinical trials [15].

## 3. CD4 Cell Differentiation, Memory, Effector Cells

**Figure 3:**
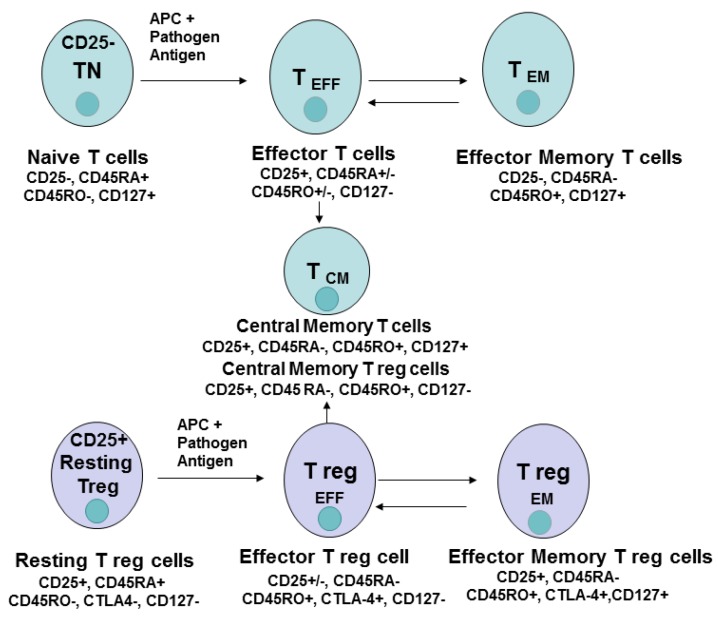
The differentiation of CD4^+^ T naive and Treg cells. The markers of each T cell type are shown during T cell differentiation. The abbreviations: TN, naive T cells; T _CM_, central memory T cells; T _EFF_, effector T cells; T _EM_, effector memory cells; Treg, regulatory T cells.

T cell differentiation and memory and effector T cells play a significant role in immunity against pathogenic agents [16]. The differentiation of CD4^+^ cells from naive to effector or memory and central memory cells is shown in Figure 3. The effector and memory cells were also demonstrated for Treg cells [16]. Once an antigen-presenting cell presents to naive T cell pathogenic antigen, T cells become activated, increase in cell number, and differentiate into effector cells which migrate to the site of infection and eliminate the pathogen. The effector cells are short-lived cells, while the subset of memory cells is formed with a potential of long-term survival-called memory cells (Figure 3). Memory cells can be located in the secondary lymphoid organs (central memory cells, T _CM_) or in the recently infected tissues––effector memory cells, T _EM_ cells (Figure 3). During re-exposure to antigen during the second immune response, memory T cells undergo fast expansion and cause more effective and faster immune response versus the primary immune response eliminating infection. The memory cells generally have several features: 1. the presence of previous expansion and activation; 2. persistence in the absence of antigen; 3. increased activity upon re-exposure to antigen [16]. The persistence of CAR-T therapy was shown to be dependent on the number of CD4^+^ cells and the number of central memory cells (CD45RO(^+^)CD62L(^+^)) in the infused product [5].

T regulatory cells differentiate into effector and memory cells. Naive conventional T cells and regulatory T cells (effector and memory subtypes) differ in their extracellular, intracellular, epigenetic, and genetic markers, transcription factors, and metabolic pathways (discussed below) (Figure 3).

## 4. CD8 Cell Subsets and Cell Differentiation

**Figure 4:**
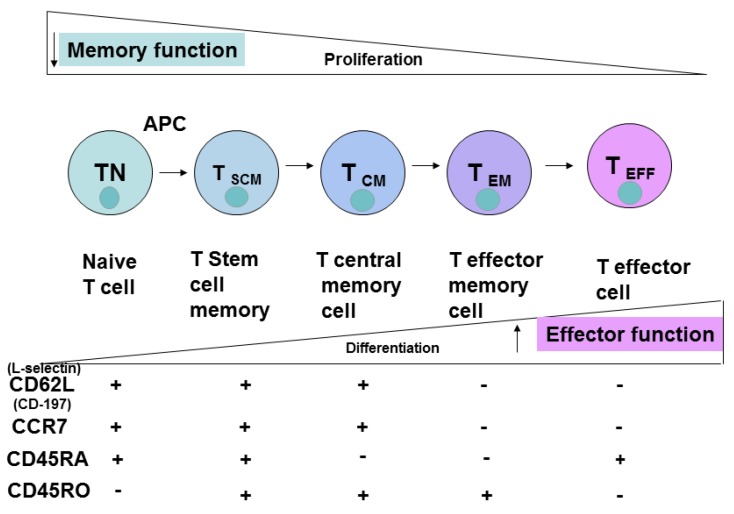
The differentiation of CD8^+^ T cells and different CD8^+^ subsets. TN, naive T cells; T _SCM_, stem cell memory T cells; T _CM_, central memory T cells; T _EFF_, effector T cells; T _EM_, effector memory cells.

The different subsets of CD8^+^ T cells are shown in Figure 4. Naive T cells differentiate into stem cell memory cells, T _SCM_; T Central Memory cells, T _CM_; T effector memory cells, T _EM_; and T effector cells, T _EFF_. The different CD8^+^ markers upon cell differentiation—L-Selectin, CD45RO, CD45RA and CCR-7—are shown in Figure 4. The effector function is increased upon CD8^+^ T cell differentiation, while memory function and proliferation are decreased (Figure 4).

CD8^+^ clones isolated from central memory T cells but not from CD8^+^ effector cells persisted long-term *in vivo* during adoptive T cell transfer in a nonhuman primate model, indicating the importance of specific T cell subset functions for effective adoptive immunotherapy [17]. Another group showed that the combination of CD8^+^ subset with CD4^+^ subset significantly enhanced T cell adoptive transfer [18]. CD4^+^ cells were shown to support development of CD8^+^ memory functions [19], demonstrating the importance of both subsets and combinations in immunotherapy trials.

## 5. Extracellular T Cell Markers

The most common phenotypic extracellular markers of naive cells are CD45RA^+^, CD45RO^−^; CD25^+^ (for Treg cells), CD62L^+^ (L-Selectin^+^), CCR-7^+^ (Figure 3 and Figure 4). These markers change to CD45RA^−^; CD62L^−^, CCR-7^−^ in CD8^+^ T_EM_ cells (Figure 4). CD45RO^−^ CD8^+^ naive cells transform into CD45RO^+^ T _EM_ cells. Thus, based on these and other phenotypic markers these cell subsets can be sorted, expanded and analyzed for functional activities during immune responses against pathogenic agents or cancer cells.

## 6. Epigenetic and Genetic Profiles

Epigenetic and genetic profiles of different subsets of T cells can be used as specific markers of each cell subtype. For example, a high level of FOXP3 transcription factor is a marker of Treg cells. For FOXP3 maintenance, the demethylation of the intronic conserved non-coding sequence 2, CNS2 is required, regulating Treg stability upon cytokine re-exposure [20]. The intronic CNS2 has been shown to be a sensor for IL-2 in Tregs and downstream target STAT-5 [20]. Key transcription factors and genetic signatures of CD8^+^ T cells during the infection were identified [21], and several clusters of key gene signatures were discovered that can predict the memory potential of CD8^+^ effector cells [21]. For example, Bcl-2 and Cdh-1 (encoding E-cadherin) were increased in the memory subset of cells.

The effector and memory T cell functions are regulated by genetic profiles of key effector genes, and also by epigenetic mechanisms such as chromatin state [22]. For example, murine memory CD8^+^ T cells are characterized by more rapid effector function upon lymphocytic choriomeningitis virus (LCMV) infection versus naïve T cells that are dependent on specific transcriptional profiles of the key effector genes *Ifng*, *Gzmb*, and *Prf1* [22]. The primary infection caused decreased nucleosomal density and less methylation of H3K27 in interferon-gamma and granzyme B chromatin that persisted in the memory stages [22] The authors proposed that these chromatin changes induced effector genes for rapid up-regulation and controlled memory functions of T cells.

## 7. Metabolic Pathways of T Cells

**Figure 5:**
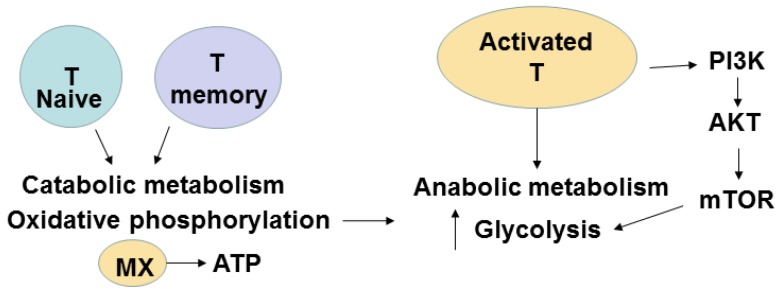
Different metabolic pathways of T cells. T naive cells and T memory cells have catabolic metabolism. T effector cells have anabolic metabolism. PI3 Kinase, PI3K; AKT and mTOR are key players of anabolic metabolism of T effector cells.

The naive and effector T cells differ in metabolic pathways. The quiescent naive T or memory cells have catabolic metabolism when nutrients are broken down to generate energy, while activated T cells have anabolic metabolism when nutrients are used to build molecular complexes and blocks to support cellular proliferation. During differentiation and activation, the metabolism is changed from oxidative phosphorylation (OXPHOS) to glycolysis [23]. The main player in anabolic pathways in activated cells is mammalian target of rapamycin (mTOR). The IL-2 and co-stimulatory CD28 signaling in activated T cells induce a switch to glycolysis with activation of PI3 kinase and downstream AKT (Figure 5). Activated AKT induces the mTOR pathway and increases utilization of glucose and amino-acids to support activated T cell proliferation. Recently, isolated metabolically active subsets of CD4^+^ and CD8^+^ T cells based on their mitochondrial membrane demonstrated increased *in vivo* persistence and anti-tumor activity [24]. In contrast to actively proliferating effector CD4^+^ and CD8^+^ T cells that depend on aerobic glycolysis with production of lactate from glucose-derived pyruvate, memory T cells have distinct metabolic pathways that depend on fatty acid oxidation [25].

Metabolic signaling varies depending on the state of T cell differentiation [26]. Th1, Th2, and Th17 CD4^+^ cells were shown to be primarily glycolytic and expressed high levels of the glucose transporter Glut1 and active mTOR, while Treg cells were dependent on lipid metabolism and had a low level of Glut1 and a high level of AMP-activated protein kinase (AMPK) [25]. Thus, the modulation of metabolic pathways is important for regulation of T cell and T cell subsets functions.

## 8. Role of Different T Cell Subsets, Treg Cells, Immune Checkpoints, Metabolic Pathways, Cytokines and T Cell Profiling in Potential Improvement of CAR-T Immunotherapy

CAR-T therapy is very effective immunotherapy against hematological malignancies [1], although many challenges exist to effectively target solid tumors [2,27,28,29,30,31]. One of the challenges is to engineer T cells to be resistant to Treg cells with suppressive signaling against tumors and to increase T cell effector and memory functions for enhanced immune response against tumors [32]. The individual patient’s T cell profile with CD4/CD8 ratio and its subsets should be analyzed and studied with the goal to improve effector and memory functions and increase the persistence of T cells for efficient CAR-T cell therapy.

Several preclinical models demonstrated the advantage of different T cell subsets for effective CAR-T therapy: CD8(+)CD45RA(+)CCR7(+) CAR-T cells with closest to the T-memory stem cells phenotype cells produced greater anti-tumor activity of CAR-T cells [6]; both CD8^+^ and CD4^+^ subsets expressed synergistic anti-tumor CAR-T activities [9]. Recent clinical trial data on patients with B cell non-Hodgkin lymphoma and chronic lymphocytic leukemia demonstrated the high anti-cancer activity of CD19-CAR-T cells generated from a composition of CD8^+^ and CD4^+^ T cell subsets that were separately expanded *in vitro* and infused at a ratio of 1:1 [33]. The same result was obtained in clinical trials on patients with B cell acute lymphoblastic leukemia [34]. These data are consistent with pre-clinical data on a combination of CD4^+^ and CD8^+^ subsets in mouse experiments [18], and CD4^+^ T cells’ role in supporting and inducing CD8^+^ T cell memory functions [19]. Another clinical study on patients with high-risk intermediate grade B-lineage non-Hodgkin lymphoma treated either with first generation CD19-CAR-T using isolated CD8^+^ T _CM_ subset or with second generation CD19-CAR-T using both CD8^+^ and CD4^+^ T _CM_ subsets demonstrated the feasibility and safety of both approaches [35], although the group of CAR-T with CD4^+^ and CD8^+^ T _CM_ and second generation CAR-T cells demonstrated better persistence. Future clinical trials using CAR-T isolated from different T cell subsets will be important for understanding the detailed mechanisms of T cell functions in CAR-T immunotherapy.

**Figure 6:**
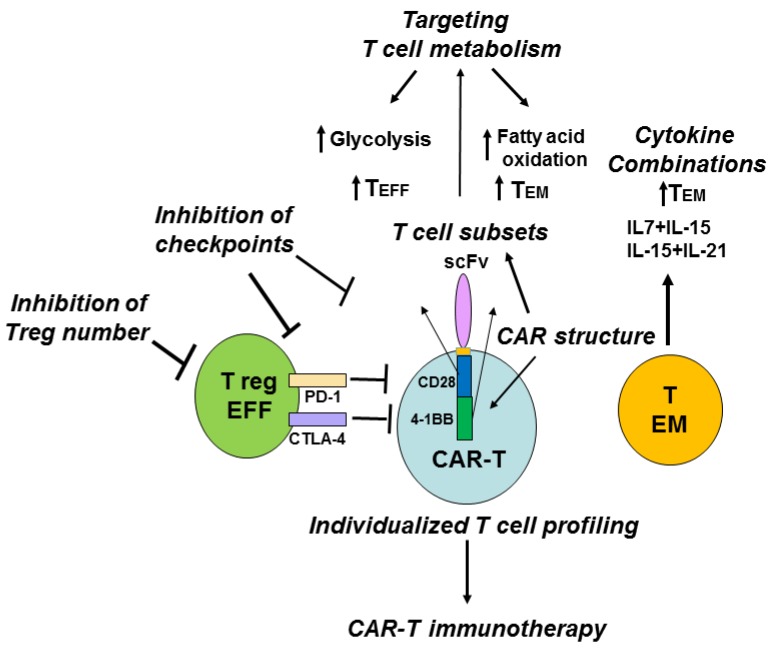
Different potential approaches to increase the efficacy of CAR-T cell therapy. The inhibition of Treg cells; inhibition of immune checkpoints such as PD-1 and CTLA-4; different T cell subsets, individualized T cell profiling; targeting T cell metabolism; combination of different cytokines and co-stimulatory CAR domains can be used to increase the efficacy of CAR-T cell therapy. Blocking PD-1 or CTLA-4 can increase efficacy of CAR-T therapy. Activation of glycolysis stimulates T_EFF_ cells, while activation of fatty acid oxidation induces T_EM_ cells. Different CAR co-stimulatory domain structure can affect T cell memory and effector functions with distinct metabolism (CAR-T cells with CD28 induce effector memory functions and glycolytic metabolism, and CAR-T with 4-1BB induce central memory and oxidative metabolism [46]) that can be applied to improve CAR-T immunotherapy.

Another aspect to consider is that the patient’s T cell profile is different from that of a healthy person and varies between the patients, suggesting that individualized T cell subset profiling and personalized immunotherapy are needed to effectively treat cancers. The development of next-generation sequencing, proteomics and metabolomics allow to create individualized immune profiles of patients and detect important T cell players that can improve CAR-T therapy (Figure 6).

It has been shown that CD4^+^ Treg cells infiltrated into solid tumors and decreased the efficacy of CAR-T therapy [36]. The authors demonstrated that anti-tumor activity of CD28-CD3ζ CAR-T cells in the presence of Treg cells was less than that of CD3ζ-CAR-T cells against tumor CEA-overexpressing tumors [36]. The CD28-CD3ζ-CAR-T cells induced infiltration of Treg cells into tumors more effectively than CD3ζ -CAR-T cells, and deletion of lck binding region inside CD28 endodomain linked to IL-2 production reversed the induction of Treg’s tumor infiltration, and increased the anti-tumor activity of CAR-T cells [36]. The administration of a high dose of Interleukin-2 was shown to increase the number of circulating CD4^+^CD25^+^Foxp3^+^ Treg cells in melanoma cancer patients [37]. The levels of CD4^+^Foxp3^+^ cells had a negative impact on adoptive immunotherapy and immune responses [38] that is consistent with the increased anti-tumor effect of CAR-T with deleted lck domain of CD28 linked to IL-2 production [36]. The result of the above study shows the IL-2-dependent antagonistic effect of Treg cells [36] versus the agonistic IL-2-dependent effect of proliferative CD8^+^ T cells on anti-tumor activity of CD28-CD3ζ-CAR-T cells demonstrated by other groups [39,40,41], and demonstrates that the balance of Treg cells to effector cells ratio is an important marker of effective immunotherapy [40]. The data with inducible T cell costimulator, ICOS-based CAR-T cells expressing Th17 profile demonstrated IL-2 independent increased persistence of these CAR-T cells *in vivo* [40]. Thus, genetic modification and structure of co-stimulatory domains of CAR-T that decreases Treg’s suppressive activity and increases the persistence and resistance of CAR-T cells can be one of the potential approaches to increase the anti-tumor efficacy of CAR-T immunotherapy (Figure 6).

Another suppressing marker that is expressed in effector Treg cells is CTLA-4 (cytotoxic T-lymphocyte-associated protein 4), which is known to block immune response and decrease the efficacy of CAR-T in pre-clinical studies [42]. To block immune checkpoints such as CTLA-4 or PD-1 (programmed cell death protein 1) with antibodies [43], or small molecules is another potential approach to increase the efficacy of CAR-T immunotherapy (Figure 6). The blockade of PD-1 immunosuppression has been shown recently to enhance CAR-T immunotherapy and to increase tumor elimination [44].

Before starting CAR-T therapy, lymphodepletion in patients using fludarabine and/or cyclophosphamide decreases the number of circulating T cells and also Treg T cells [45]. The lymphodepletion induces proliferation of transferred T cells by decreasing competition for interleukins-7 and 15 which support proliferation of pre-existing T cells. Thus, more efficient expansion of transferred T cells rather than preexisting T cells enhances CAR-T immunotherapy. Recent clinical trials demonstrated the efficacy of lymphodepletion approaches in improving CAR-T immunotherapy [34,35].

An alternative potential approach is to target T and CAR-T cell metabolism and thus increase effector cell functions, for example, by switching from catabolic to anabolic metabolism (Figure 6). In the murine asthma model, stimulation of Treg metabolic player, AMP-activated protein kinase, was sufficient to decrease Glut1 and increase Treg generation [25]. Targeting T cell metabolism may stimulate immunity by promoting the glycolytic metabolism pathway characteristic of T _EFF_ cells or suppress immunity and inflammation by promoting the lipid oxidation characteristic of Treg cell subsets [26]. Recently, a different role of co-stimulatory CAR domains 4-1BB or CD28 has been demonstrated in regulation of specific metabolism pathways and memory functions of CAR-T [46]. The 4-1BB domain inside CAR stimulated growth of CD8(+) central memory T cells with elevated respiratory capacity, increased fatty acid oxidation, and enhanced mitochondrial biogenesis, while CD28 domain inside CAR induced effector memory cells with molecular profile of enhanced glycolysis [46]. Thus, modulation of metabolism pathways by either metabolic inhibitors or by using a different structure of co-stimulatory domains and receptors can modulate immune response with desired balance of short-lived effector and long-lived memory cells to improve CAR-T immunotherapy (Figure 6).

The modulation of cytokine cocktails can change the differentiation status of T cells [47]. For example, a combination of IL-12 plus IL-7 or IL-21 for 3 days with withdrawal of IL-12 led to a less differentiated T cell phenotype (CD62L^+^, CD28^+^,CD27^+^, CD127^+^, CCR-7^+^) and to up-regulation to stem cell markers such as Nanog, SOX-2, Oct-4, and LIN28A [47]. Pre-treatment of T cells with IL-7 and IL-15 or IL-15 and IL-21 was shown to increase T memory cell functions and anti-tumor activity of CAR-T cells [6,48]. IL-15 has been shown to increase CD8^+^ T memory cell function and increase T cell anti-tumor activity [49]. CAR-T cells expanded with IL-7+IL-15 had higher survival *in vivo* compared with CAR-T expanded with IL-2 [6]. Culturing CD3/CD28-CAR-T in the presence of IL-7 and IL-15 gave the best effector activity while retaining a stem/memory against GD2 tumor antigen [50]. Thus, modulation of interleukin cocktails can affect the memory functions of T cells that can be used as an alternative potential approach to increase the efficacy of CAR-T immunotherapy (Figure 6).

## 9. Conclusions and Perspectives

This report shows the complexity of T cell differentiation, stem cell memory, memory and effector functions, their regulatory, intracellular, extracellular markers, cellular signaling, metabolism, cytokine-directed regulation of T cell differentiation and function that should be considered during cellular immunotherapy, including CAR-T therapy. Since CAR-T therapy includes withdrawal of T cells from cancer patients used for expansion, the ratio of CD4/CD8, and memory markers such as CD45RA, CD45RO and other markers such as CD26, CD95, CCR-7, and CD62L should be analyzed for a selection of different T cell subsets at different ratios to be studied in clinical trials for more effective and personalized therapy. The sorted CD4 or CD8 cell populations and T cell subsets with specific memory or stem cell memory markers and genetic, epigenetic, and metabolic profiles in combination with immune checkpoint inhibitors should be analyzed and used for regulating the efficacy of CAR-T therapy. The modulation of metabolic pathways of different T cell subsets with metabolic inhibitors will provide novel mechanisms of CAR-T immunotherapy. The genetic modifications of CAR constructs and the effects of the modification of inhibitory and stimulatory pathways on the efficacy on CAR-T cells can be studied further. Future studies with personalized T cell subsets patient profiles and the above discussed approaches will illuminate the key mechanisms of efficient CAR-T therapy against hematological malignancies and solid tumors.
